# The protective role of tissue-resident interleukin 17A–producing gamma delta T cells in Mycobacterium *leprae* infection

**DOI:** 10.3389/fimmu.2022.961405

**Published:** 2022-10-26

**Authors:** Yan Liu, Chao Shi, Shanshan Ma, Yuelong Ma, Xinyuan Lu, Jianyu Zhu, Degang Yang

**Affiliations:** ^1^ Department of Infectious Diseases, Shanghai Skin Disease Hospital, Tongji University School of Medicine, Shanghai, China; ^2^ Shanghai Key Laboratory of Pathogenic Fungi Medical Testing, Shanghai Pudong New Area People’s Hospital, Shanghai, China

**Keywords:** leprosy, gamm delta T cell, IL-17A, dermis, IL-23, ROR

## Abstract

Mycobacterium *leprae* is a kind of disease-causing bacteria and results in leprosy in human. Gamma delta (γδ) T cell is a T-cell subset that is presented in both human dermis and epidermis. These cells bridge innate and adaptive immune responses and play critical roles in regulating anti-microbial defense, wound healing, and skin inflammation. Here, we investigated skin resident γδ T cells in patients with leprosy. Our data showed that γδ T cells significantly accumulated in skin lesions of leprosy patients with tuberculoid (TT) form. IL-23 can predominantly stimulate dermal γδ T cells to produce interleukin 17 (IL-17), a cytokine which may lead to disease protection. These γδ T cells expressed a specific set of surface molecules, and majority of these cells were Vδ1^+^. Also, IL-23 can stimulate the expansion of dermal γδ T cells expansion. Moreover, our results revealed that the transcription factor RORγt was responsible for IL-17A expression in leprosy lesion. Therefore, these data indicated that IL-23–responsive dermal γδ T cells were the major resource of IL-17A production in the skin and could be a potential target in the treatment of leprosy.

## Introduction

The infection of the bacillus Mycobacterium *leprae* (M. leprae), which results in tissue destruction and demyelination of peripheral nerves, is the cause of the chronic infectious disease known as leprosy (1, 2). It is generally believed that the disease has a high infectivity but a low pathogenicity. Clinically, the infection of M. leprae results in intense instability in the immune system of humans, which may reflect the spectrum of the disease. Patients who have the tuberculoid (TT) form experience a cell-mediated reaction that is defined by the release of inflammatory cytokines (3–5).

Interleukin 17A (IL-17A) is generally considered as a pro-inflammatory cytokine. IL-17A has been shown to play a protective role in the early stages of infection by triggering macrophages and boosting Th1 effector cells (6, 7). Saini et al. discovered CD4^+^ Th17 cells in the patients infected with Mycobacterium tuberculosis and emphasized the significance of these cells in leprosy. Also, they discovered that, in leprosy patients with TT form, skin lesions and peripheral blood mononuclear cell (PBMC) preparations activated with M. leprae antigen had greater expression and release of IL-17A (8).

Gamma delta (γδ) T cells are a broad subtype of unconventional T cells. These cells have T-cell receptor (TCR), which is composed of one γ (gamma) chain and one δ (delta) chain (9), and can secrete cytokines such as interleukin 17 (IL-17), interferon γ (IFN-γ), and IL-22 (10–12). γδ T cells are generally accumulated in barrier tissues including the reproductive tract, gastrointestinal tract, and skin but with low numbers in peripheral blood (13–17). Human γδ T cells mainly exist in the dermis (18, 19), whereas a small portion can be found in the epidermis in steady state (20). Studies have shown that circulating γδ T cells express δ2 TCR, whereas skin resident cells express δ1 TCR (21–23). γδ T cells are involved in both innate immune responses and adaptive immune responses and play critical roles in different diseases.

Recently, researchers have started to explore the numerous functions of γδ T cells in anti-microbial defense and skin inflammation. These cells may play a key role in the response to various pathogens in the skin (15, 24, 25). Although it has been reported that circulating γδ T cells in peripheral blood are increased in leprosy patients with reversal reactions (RRs) and erythema nodosum leprosum (ENL), it is still unclear whether this population could be involved in lesional response to M. lepra. In this study, we showed that γδ T cells were enriched in leprosy lesion. These cells were the major IL-17A–excreting cell type in the skin of leprosy patients after IL-23 stimulation and had a unique surface phenotype. More importantly, we confirmed that γδ T cells and IL-17A secreted by these cells under the regulation of RORγt were crucial for leprosy protection *in situ*. These results indicated that IL-17A–producing dermal γδ T cells were one of the key components in the protection of TT leprosy.

## Results

### Leprosy lesions were enriched with γδ T cells

**Figure 1:**
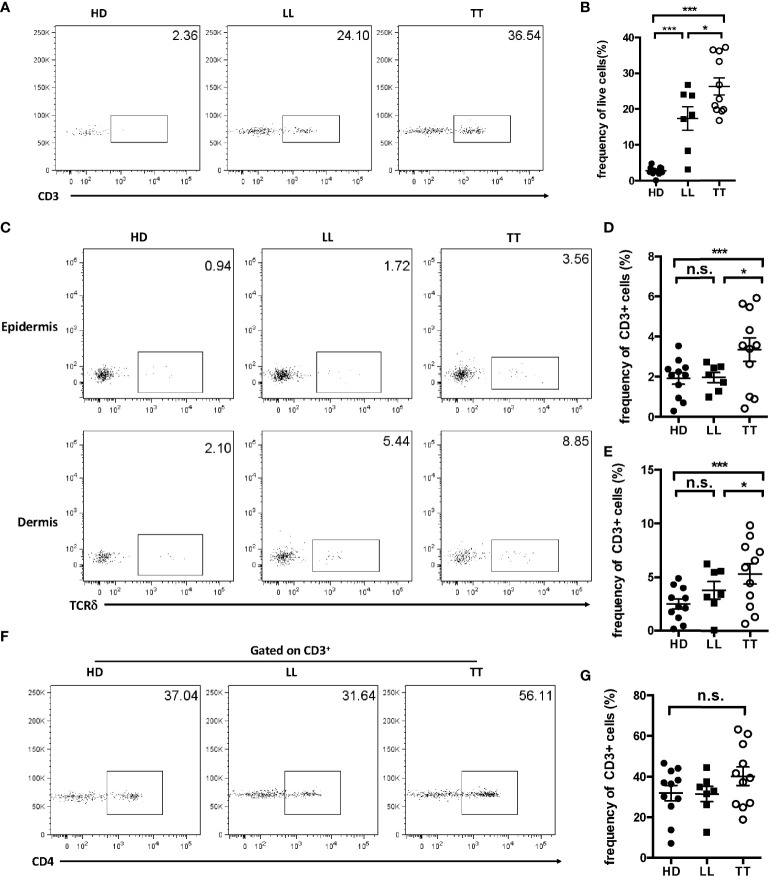
Enrichment of γδ T cells in leprosy lesions. **(A)** Dermal cells from healthy controls and skin lesion of leprosy patients were analyzed for CD3 by flow cytometry. **(B)** Statistical results in **(A)**. **(C)** Epidermal and dermal cells from healthy controls and skin lesion of leprosy patients were analyzed for γδ T cells by flow cytometry. **(D)** Statistical results in **(C)**. **(E)** Dermal cells from healthy controls and skin lesion of leprosy patients were analyzed for CD4 by flow cytometry. **(F)** Cells from healthy controls and leprosy patients were analyzed for CD4+ T cells by flow cytometry. **(G)** Statistical results in **(F)**. Flow plots gated on live cells were representative from 11 TT patients, seven LL patients, and 11 healthy donors. Percentage was shown as mean ± SEM. Statistical analysis was performed by a one-way ANOVA or *t* test. n.s.: no significance; *p < 0.05; ***p < 0.001.

Skin resident γδ T cells participate in the development of inflammatory skin diseases, autoimmune skin diseases, and skin tumors. However, the role of resident γδ T cells in leprosy had not been clearly clarified. We found that more T lymphocytes were infiltrated into skin with leprosy both in TT and LL forms than controls (**Supplementary Figure S1**). To determine whether γδ T cells exist in leprosy lesions, we examined this population in healthy donors, TT leprosy patients, and lepromatous (LL) leprosy patients and found that the frequency of CD3^+^ T lymphocyte infiltration in lesions of TT and LL leprosy patients were both much higher than that in healthy skin by flow cytometry (**Figures 1A, B** **)**. Further analysis showed that the fraction of γδ T cells in total Tlymphocytes increased significantly in TT lesions compared with the counterpart in control group but not in LL group (**Figures 1C–E**). In contrast, there was a little difference in the frequency of CD4 T cells between healthy skin and TT and LL leprosy patients, although there was indeed an increase tendency in TT leprosy patient group, which may be due to limited samples in this study (**Figures 1F, G** **)**.

### Dermal γδ T cells were the major source of IL-17A in skin

**Figure 2:**
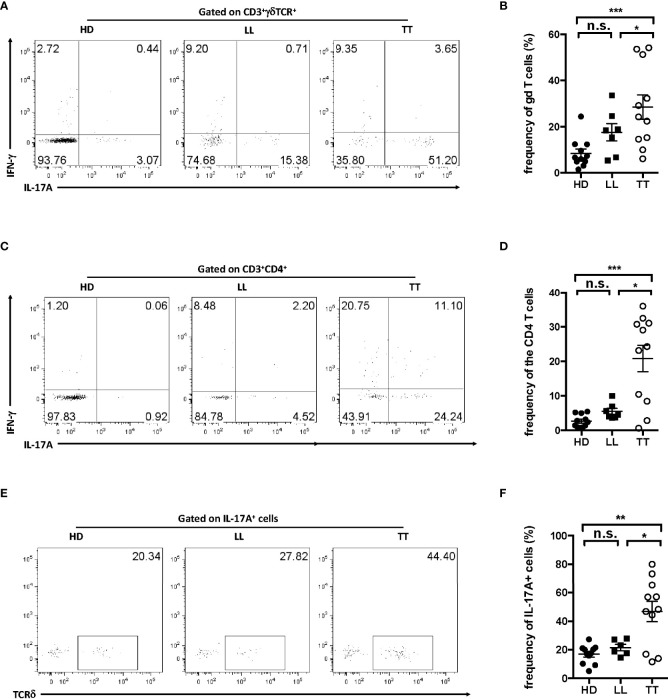
Dermal γδ T cells were predominant IL-17–producing cells in leprosy lesion. Intracellular IL-17A production was assessed by flow cytometry on dermal cell suspensions from leprosy lesion and healthy skin. **(A)** Representative plots showed expression of IL-17A and IFN-γ in γδ T cells. **(B)** Statistical results in **(A)**. **(C)** Representative plots showed expression of IL-17A and IFN-γ in CD4 T cell. **(D)** Statistical results in **(C)**. **(E)** Representative plots showed TCRδ^+^ cells among IL-17A^+^ cells. **(F)** Statistical results in **(E)**. Percentage was shown as mean ± SEM. Statistical analysis was performed by *t*-test. n.s.: no significance; *p < 0.05; **p < 0.01; ***p < 0.001.

IL-17A has been clearly linked to the pathogenesis of inflammatory skin disease; thus, to further detect IL-17A–producing cells in leprosy skin lesion, single-cell suspensions were prepared from dermis. IL-17A^+^ cells accounted approximately 8% in dermal live cells from LL patients and 25% in that from TT patients (**Supplementary Figure S2**), suggesting an important role of this cytokine in the disease. Dermal γδ T cells were approximately 30% IL-17A^+^ in TT leprosy and in LL were nearly 20% (**Figures 2A, B** **)**, whereas the fraction for CD4^+^ T cells was only about 20% in TT groups with even lower percentage in LL groups (**Figures 2C, D** **)**, indicating higher fraction of IL-17A^+^ in γδ T cells than in CD4 T cells. To further determine which subset produced more IL-17A in skin, we examined the proportion of γδ T cells in IL-17A^+^ cells and found that more than 50% IL-17^+^ lymphocytes were TCRδ^+^ in TT patients, and comparable level of TCRδ ^+^ cells between LL patients and healthy controls (**Figures 2E, F** **)**, indicating that dermal γδ T cells were the major source of IL-17A in dermis from lesion of TT but not of LL leprosy. Therefore, further analysis in this study, we focused on lesion of TT leprosy, as IL-17A^+^ γδ T cells in this group showed more significantly enrichment in lesions.

### IL-17A–producing γδ T-cell proportion negatively correlated with nerve dysfunction

**Figure 3:**
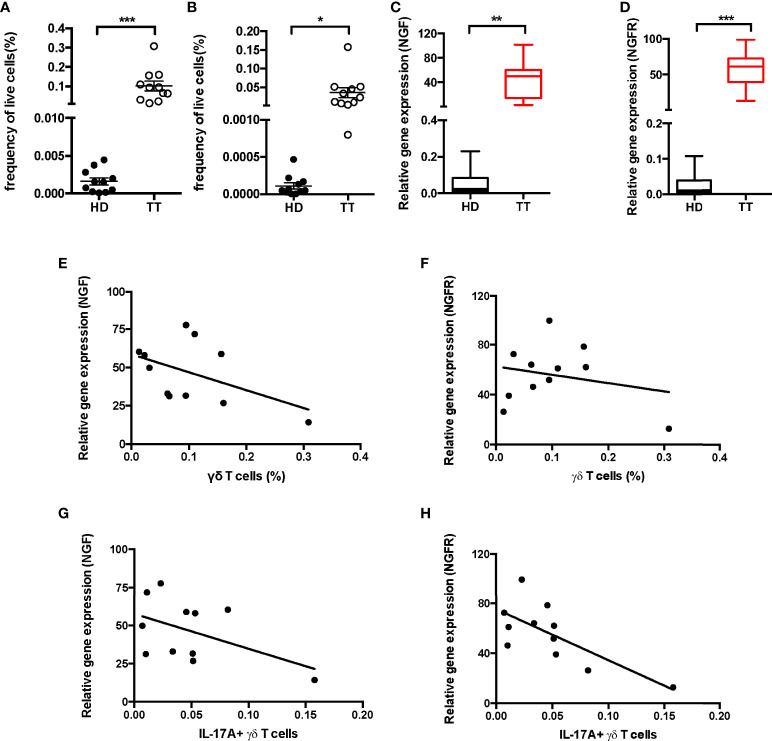
Correlation analysis between IL-17A^+^ γδ T cells and NGF, NGFR in leprosy lesion. Quantification of γδ T cells **(A)** and IL-17A^+^ TCRδ<σπ>+</σπ> cells **(B)** in dermis from 11 patients and 11 healthy donors. Expression of NGF **(C)** and NGFR **(D)** mRNA measured by qPCR in leprosy lesions or healthy skin. Correlation analysis between fraction of dermal γδ T cells and NGF **(E)** and NGFR **(F)** expression or fraction of dermal IL-17A^+^ TCRδ^+^ cells and NGF **(G)** and NGFR **(H)** expression. Percentage was shown as mean ± SEM. Statistical analysis was performed by *t*-test. *p < 0.05; **p < 0.01; ***p < 0.001.

To reflect the dysfunction of nerve in the development of leprosy, changes in the production of nerve growth factor (NGF) and the receptor were adopted to assess the disease severity, as they could be directly correlated with sensory loss and disability. To test whether γδ T cells were responsible for severity of nerve dysfunction, we quantified the proportion of dermal γδ T cells (**Figure 3A**) and IL-17A^+^ TCRδ<σπ>+</σπ> cells (**Figure 3B**) from patients with TT leprosy by flow cytometry. In addition, we determined the levels of NGF (**Figure 3C**) and NGFR (**Figure 3D**) in leprosy lesion using real-time polymerase chain reaction (PCR). Data showed that the expression of NGF and NFGR increased more robustly in patients with TT leprosy than in HD.

Furthermore, we examined the association between the proportion of lesion γδ T cells and expression of NFG or NGFR and did not find any significant correlation (**Figures 3E, F** **)**. In contrast, the expressions of NGF and NGFR were negatively correlated with the proportion of dermal IL-17A–producing γδ T cells (**Figures 3G, H** **)**, indicating negatively regulation of IL-17A^+^ γδ T cells on NGF and NGFR expression in lesion of TT leprosy.

### TCR chain usage of IL-17A–producing dermal γδ T cells

**Figure 4:**
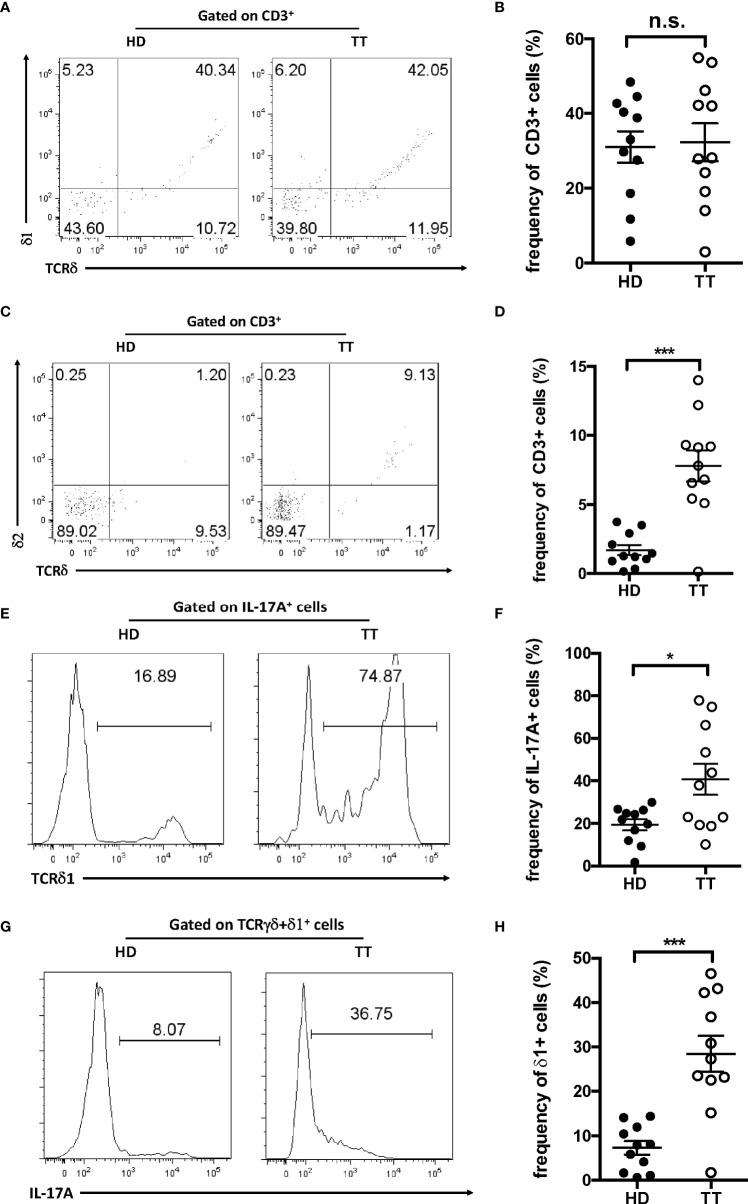
TCR usage of dermal γδ T cells. Dermal cell suspensions were stained with Vδ1 **(A)** and Vδ2 **(C)** and analyzed with flow cytometry. Flow plots gated on CD3^+^ cells. **(B)** Statistical results in **(A)**. **(D)** Statistical results in **(C)**. **(E)** Histogram of Vδ1^+^ cells among IL-17^+^ dermal cells from leprosy lesions or healthy skin. **(F)** Statistical results in **(E)**. **(G)** Histogram of IL-17A–producing cells among dermal Vδ1^+^ cells from leprosy lesions or healthy skin. **(H)** Statistical results in **(G)**. Percentage was shown as mean ± SEM. Statistical analysis was performed by *t*-test. n.s.: no significance; *p < 0.05; ***p < 0.001.

To determine whether dermal γδ T cells in TT leprosy skin lesion used a unique γδ TCR, we first evaluated the TCR delta usage through a panel of antibodies specifically for V delta TCR segments including Vδ1 and 2. As depicted in **Figures 4A, B**, almost 30% of dermal γδ T cells expressed Vδ 1 both in lesions of TT leprosy patients and in healthy skin (**Figures 4A, B** **)**. Vδ2 was only detected on a small fraction in lesions of TT leprosy patients but not of healthy controls (**Figures 4C, D** **)**. In addition, approximately 50% IL-17A–producing dermal γδ T cells expressed Vδ1 (**Figures 4E, F** **)**; however, only a fraction of Vδ1 γδ T cells were IL-17A–producing γδ T cells (**Figures 4G, H** **)**. These results indicated that dermal γδ T cells showed a unique TCR Vδ usage with IL-17–producing capability. Compared proportion of Vd1 and Vd2 in lesions from lesions of TT patients to healthy skin, comparable level of Vδ1 and increased level of Vδ2 suggested that majority of the dermal γδ T cells in leprosy lesion were from original expansion but not infiltrated from peripheral blood.

### IL-17A–producing dermal γδ T cells were phenotypically unique

**Figure 5:**
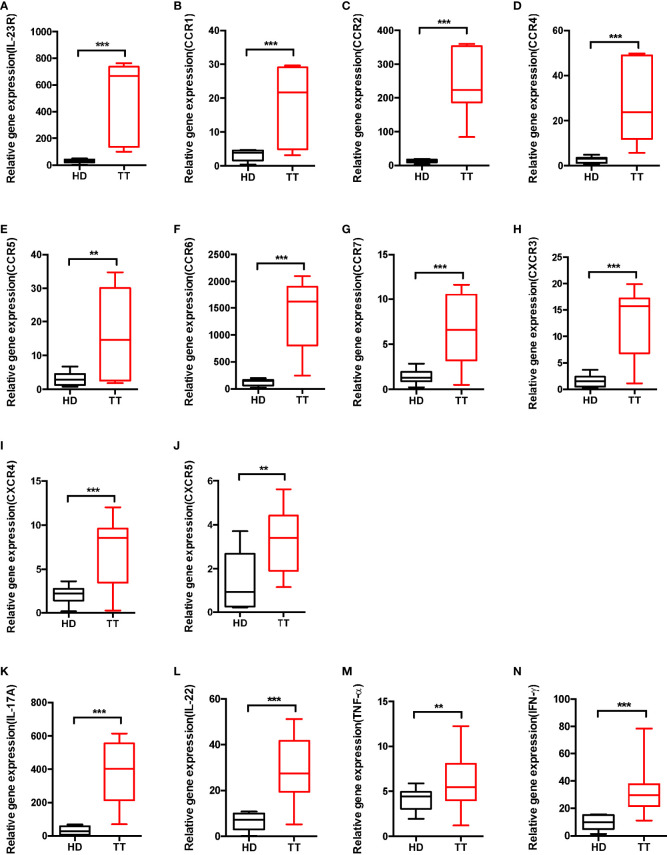
Phenotypic analysis of dermal γδ T cell in leprosy lesions. **(A–J)** Expressions of IL-23R and chemokine receptors mRNA were measured by qPCR. **(K–N)** mRNA expression levels of cytokine produced by dermal γδ T cells were measured by qPCR. Relative gene expression indicated gene normalized for b-actin and was shown as mean ± SEM. Statistical analysis was performed by *t*-test. **p < 0.01; ***p < 0.001.

To further determine the surface expression pattern, we performed real-time PCR to detect the mRNA level of chemokine receptor and IL-23R. As presented in **Figure 5**, dermal γδ T cells expressed various chemokine receptors such as CCR1, CCR2, CCR4, CCR5, CCR6, CCR7, CXCR3, CXCR4, and CXCR5, and constitutively IL-23R (**Figures 5A–J**).

To further evaluate whether dermal γδ T cells produce other cytokines such as IL-22 and TNF-α, γδ T cells from skin were stimulated *ex vivo*. Although there were differences in production of IL-22, TNF-α and IFN-γ between lesions of TT leprosy and healthy controls, skin dermal γδ T cells mainly produced large amount of IL-17A (**Figures 5K–N**). Thus, dermal γδ T cells appeared to be developmentally skewed toward IL-17A–producing cells.

### IL-23 stimulated *ex vivo* expansion of dermal γδ T cells

**Figure 6:**
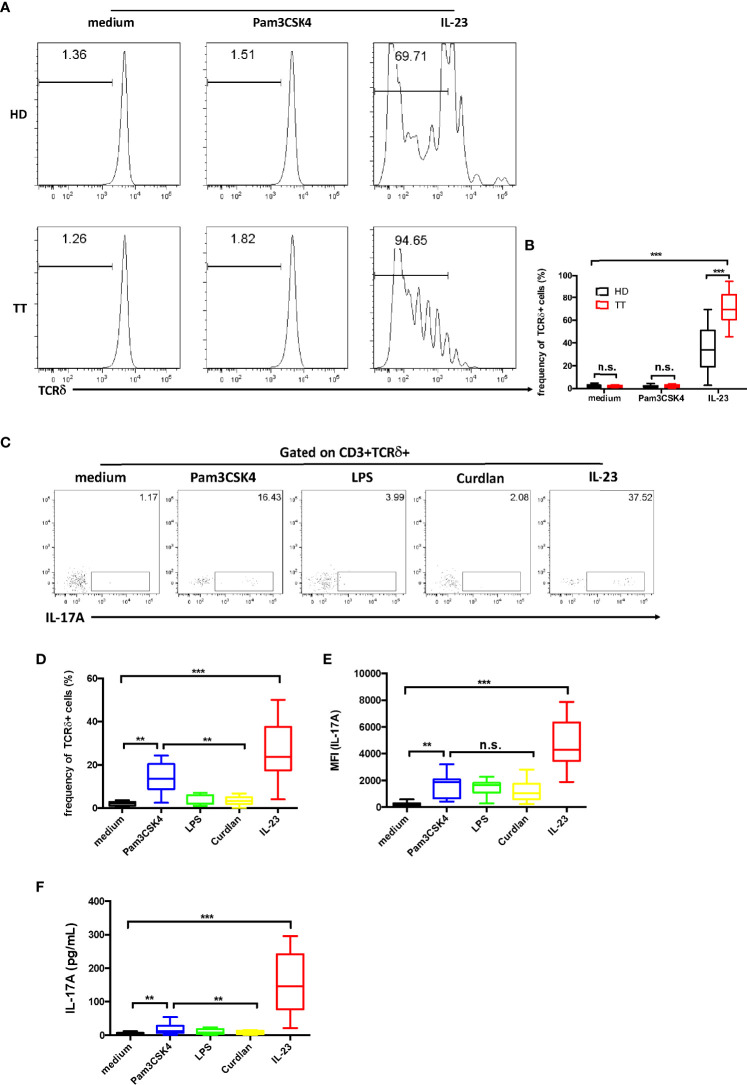
Dermal γδ T cells *ex vivo* expansion and IL-17A production upon IL-23 stimulation. **(A)** Dermal cells were labeled with Carboxyfluorescein succinimidyl ester (CFSE) and then stimulated with Pam3CSK4 or IL-23 for 4 days. Cells were analyzed by flow cytometry with CD3 and TCRδ staining. **(B)** Statistical results in **(A)**. **(C)** Dermal cell suspensions were stimulated with Pam3CSK4, LPS, Curdlan, or IL-23 for 48h. IL-17A production was determined by flow cytometry. **(D, E)** Statistical results in **(C)**. **(F)** Supernatants harvested from **(B)** were examined for IL-17A secretion by ELISA. Statistical analysis was performed by two-way ANOVA or one-way ANOVA. n.s.: no significance; **p < 0.01; ***p < 0.001.

Previous researches have shown that the TLR2 agonist Pam3CSK4 could activate murine splenic γδ T cell expansion *in vitro*, whereas IL-23 does not have this effect (26). We therefore stimulated skin cells with Pam3CSK4 or IL-23. Data revealed that Pam3CSK4 alone did not stimulate dermal γδ T cell proliferation. However, we identified that IL-23 alone was sufficient for promoting the proliferation of dermal γδ T cells (**Figures 6A, B** **)**.

We next evaluated whether these pathogen products could affect IL-17 production of the dermal γδ T cells. By intracellular cytokine staining, we found that TLR agonists Pam3CSK4 (TLR2) stimulated dermal γδ T cells to produce low amount of IL-17, whereas lipopolysaccharide (LPS) (TLR4) or dectin-1 ligand curdlan did not show this capacity (27) (**Figures 6C, D** **)**. However, the data of the mean fluorescent intensity (MFI) revealed an increase of IL-17 production by the stimulation of IL-23 (**Figure 6E**). Moreover, when cells were exposed to IL-23, the data from enzyme-linked immunosorbent assay (ELISA) analysis showed a robust increase on IL-17 production (**Figure 6F**).

These data suggest that IL-23 is critical in both regulating γδ T cell differentiation and maintaining homeostasis of these cells.

### RORγt promoted IL-17A production, leading to γδ T cell differentiation

**Figure 7:**
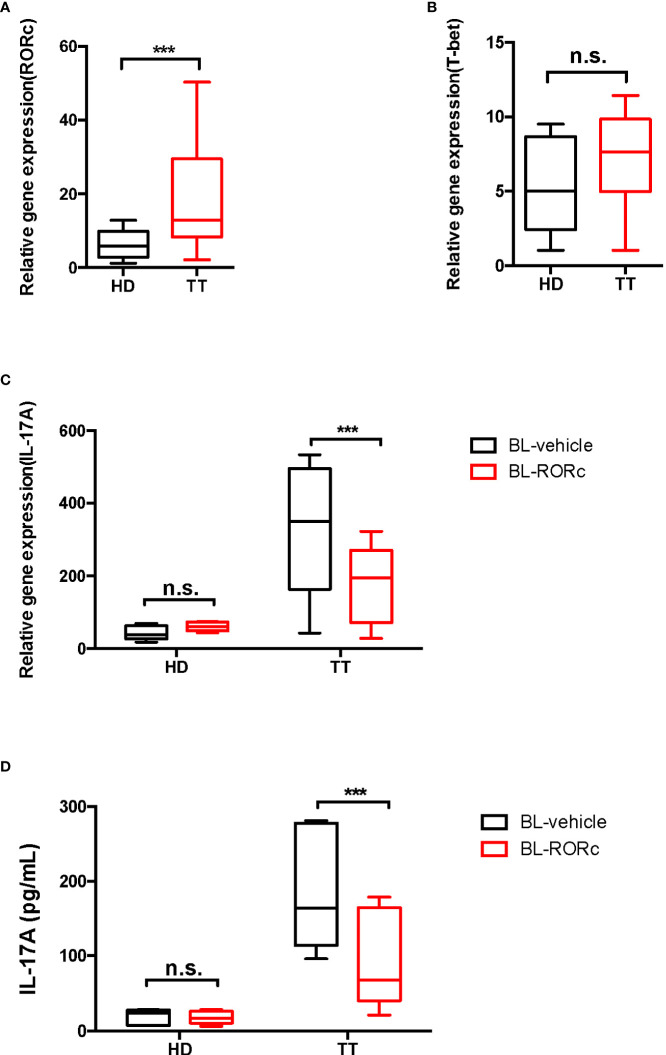
RORγt gene silencing suppressed the production of dermal γδ T cells in leprosy lesions. mRNA expression level of *RORc* **(A)** and *T-bet* **(B)** in dermal γδ T cells were determined by qPCR. Relative gene expression indicated gene normalized for β*-*actin. **(C)** qPCR was performed to measure the transcript levels of *Il17a* in BL-RORc^△^ or BL-vehicle dermal γδ T cells. **(D)** ELISA was performed to measure the secretion of IL-17A in BL-RORc^△^ or BL-vehicle dermal γδ T cells. Data were shown as mean ± SEM. Statistical analysis was performed by *t*-test or two-way ANOVA. n.s.: no significance; ***p < 0.001.

Based on the reported transcriptional profiles (28), transcription factors such as T-bet and RORγt, which are typically associated with γδ T cells, revealed greatly elevated levels in γδ T cells from leprosy lesions. As confirmed by qPCR, the expression of *RORc*, a factor that promotes IL-17A production was elevated exclusively in lesion γδ T cells (**Figure 7A**). In contrast, *T-bet* expression was not markedly affected (**Figure 7B**). Thus, we mainly focused on RORγt, and silenced this transcription factor in γδ T cells by RNAi. The efficiency of this system was determined by *RORc* qPCR (**Supplementary Figure S3**).

To determine the regulatory role of RORγt in IL-17A expression, we cultured suspensions of cells from the lesion of leprosy patients *ex vivo. RORc* expression was knocked down in γδ T cells using RNAi and IL-17A expression was measured through qPCR and ELISA. We found that levels of IL-17A decreased significantly in the BL-RORc^△^ group (**Figures 7C, D** **)**.

## Discussion

In this study, we identified that γδ T cells were accumulated in the skin lesions of leprosy patients with TT form and had a unique surface phenotype. These dermal populations were the major source of IL-17A under the regulation of RORγt. IL-23 preferentially stimulated the proliferation of dermal γδ T cells, indicating a potential feed-forward mechanism in leprosy. Thus, dermal γδ T cells were critical IL-17A–producing cells in protection of TT leprosy.

IL-17 producing CD4^+^ T cells have been considered as an independent subtype of helper T cells, which contribute to maintain the stability of leprosy (8) and promote inflammation in leprosy reactions (29). IL-17 producing CD4^+^ T cells have been reported in other bacterial and viral infections such as tubercle bacillus, leishmaniasis, and HIV (30–32) and present pathogenesis in the experimental models of autoimmune diseases (33). Studies have shown that IFN-γ–producing Th1 cells can control the infection of M. *leprae* (34). However, Th1 cells alone do not fully explain the resistance or susceptibility to infections and diseases (35, 36), indicating that other factors should be required for the regulation of immune status and disease progression of leprosy (37). In addition, it has been reported that Th1 and Th17 cells are dysregulated in the infection of Mycobacterium (38). In this study, we demonstrated an additional T-cell subtype, TCRγδ T cells, which could excrete IL-17 in response to the cytokine IL-23 in the skin lesion of leprosy patients. Also, TCRγδ^+^IL-17A^+^ cell increased in TT leprosy lesion compared with the healthy control group.

Similar to Th17 cells, IL-17A–producing γδ T cells express RORγt (39). RORγt is a transcription factor from retinoic acid–related orphan nuclear receptor (ROR) family, and it could regulate *Il17a* transcription by binding RORE sequences presented in the upstream of the promoter (40). In addition, it could also bind the conserved non-coding sequences (CNS)2 of *Il17a* gene (41, 42). Thus, RORγt is associated with IL-17A production from γδ T cells (43). Moreover, γδ T cells maintain a high-constitutive RORγt expression level than other T cells (44). There was a defect in IL-17A production in RORγt knockout mice (43). Taken together, RORγt could promote the differentiation of IL-17A–producing γδ T cells.

IL-23/IL-17A axis plays an important role in skin inflammation and autoimmune diseases, including psoriasis (45, 46). In mice, Cai et al. showed IL-23 could promote IL-17A secretion by γδ T cells (15). For Th17 cells, IL-23 was necessary for the maintenance of these cells (47). It was reported that the expansion of splenic γδ T cell *ex vivo* could be stimulated through TLR2 signaling (26). In this work, we found that there was a higher fraction of γδ T cells in *ex vivo* culture with IL-23 than with Pam3CSK4, indicating similar effect of IL-23R signaling in IL-17A–producing γδ T cells compared with Th17 cells.

The majority of human γδ T cells expresses three subtypes of d chain, including Vδ1, Vδ2, and Vδ3 (48). Vδ1^+^ cells account 30% of peripheral γδ T cells and are also found in gut epithelium and other organs. Typically, this chain could associate with different γ chain as Vγ2, Vγ3, and Vγ4. Vδ2<σπ>+</σπ> cells are the main subset in healthy individuals. It constitutes more than 50% of γδ T cells in PBMC. These cells could be divided into Vγ9^+^ and Vγ9^-^ subsets. Both Vδ1^+^ and Vδ2^+^ cells could produce IL-17A and IFN-γ (49, 50). There were some studies demonstrated that Vδ1^+^ cells were involved in several diseases (51–54). These two γδ T-cell subsets could both be detected in leprosy lesion; however, their roles were still yet to be deciphered.

For IFN-γ–producing γδ T cell, they were also upregulated in leprosy lesion of TT form as compared with healthy controls. This was consistent with previous findings that IL-17, IFN-γ, and Foxp3 levels from PBMC showed increase in patients with leprosy reactions compared with non-reactions leprosy patients (29). In patients with leprosy reactions, the level of IFN-γ was high due to cell-mediated immune responses and immune complex. However, the role of IFN-γ in IL-17 excretion by γδ T cells is not fully understood in M. *leprosy* infection. Here, we identified the expression of IL-17 and IFN- γ from γδ T cells in a reciprocal manner and higher in both leprosy reactions.

There are some limitations in this study. Our data showed that both IFN-γ^+^ and IL-17A^+^ γδ T cells in leprosy skin lesion significantly increased compared with healthy controls. It is possible that IFN-γ^+^ and IL-17A^+^ γδ T cells share some common features with other human infections and autoimmune diseases in skin, and further investigation is necessary to prove this hypothesis. Moreover, in *ex vivo* experiments, gene expressions were detected only in transcriptional level; flow cytometry or other protein detection should be performed to confirm these findings. Also, the IL-17A–producing cells in RNAi analysis need to be confirmed by flow cytometry. Further studies will help elucidate the functional roles of IL-17–producing γδ T cells.

In conclusion, we demonstrated the role of IL-17A–producing γδ T cells in the lesion of TT leprosy. These cells were negatively correlated with disease severity and suggested potentially protective functions in TT leprosy patients.

## Material and method

### Patients and ethics

Patients with TT and LL forms were diagnosed based on the clinical and histopathologic criteria. Skin biopsies were collected from the lesion site of 11 tuberculoid leprosy, seven lepromatous leprosy, and 11 normal volunteers. The Ethics Committee of Shanghai Skin Disease Hospital approved this research. All the participants gave their written informed consent.

### Skin cell preparation

These processes were performed as previous described (55). Briefly, human skin samples from patients with TT or LL leprosy and from healthy donors were collected and incubated for 2h at 37°C with 1 mg/ml collagenase IV (Sigma-Aldrich, St. Louis, MO), 0.4 mg/ml hyaluronidase (Sigma-Aldrich), and 0.03 mg/ml DNase-I (Sigma-Aldrich) in RPMI 1640 medium (supplemented with 5% fetal bovine serum and 100 U/ml penicillin-streptomycin [Invitrogen, Camarillo, CA]). After filtration through a 70-μm cell strainer (BD Bioscience, San Jose, CA), a single-cell suspension was obtained.

Mononuclear cells were isolated from skin-cell suspensions using density gradient centrifugation with a Lymphoprep solution (AXIS-SHIELD, Oslo, Norway) and were resuspended in RPMI 1640 (Invitrogen).

### *Ex vivo* stimulation

0.2 × 10^5^ cells/100 μl dermal from HD or leprosy patients were incubated in RPMI-1640 (Invitrogen), 2 mg/ml recombinant IL-23 (R&D system, Minneapolis, MN), 1 mg/ml Pam3CSK (Merck, Rahway, NJ), 100 ng/ml LPS (Merck) or 50 mg/ml Curdlan (Merck) was added at a concentration of 2 mg/ml for 24h, the supernatant was harvest for IL-17A measurement by IL-17A Human ELISA kit (Thermo Fisher Scientific, Waltham, MA) according to manufacturer’s instructions, then stimulated with presence of GolgiPlug (BD Bioscience) for 4h.

### Flow cytometry analysis and intracellular staining

Human CD3 (BD Bioscience), CD4 (BD Bioscience), TCRgd (BD Bioscience), TCR Vd1 (Invitrogen), TCR Vδ2 (Invitrogen), IL-17A (BD Bioscience), and IFN-γ (BD Bioscience) mAbs were purchased from BD bioscience or Thermo Fisher Scientific. In brief, for cell surface staining, 0.5 × 10^5^ cells/100 μl staining buffers were incubated with a cocktail for 30 min at 4°C. For intracellular staining, cells were fixed and permeabilized (BD Bioscience) and then stained intracellularly for IL-17A and IFN-γ. Flow cytometry analysis was performed on BD FACS Calibur (BD Bioscience) and analyzed with FlowJo software (Flowjo LLC, Ashland, OR).

### *In vitro* proliferation assays

Dermal cells suspensions were labeled with CFSE (Sigma-Aldrich) and then stimulated with 1 mg/ml of Pam3CSK (Merck) or 2 of mg/ml recombinant IL-23 (R&D system) for 4 days. Cells were harvested and stained with CD3 and TCRγδ. Proliferation was measured by expression of CFSE (Sigma-Aldrich).

### RNA extraction and real-time quantitative PCR

RNAs were isolated with a QIAWAVE RNA Mini kit (QIAGEN, Dusseldorf, Germany). After reverse transcription into cDNA with a Reverse Transcription Kit (Bio-Rad, Hercules, CA). Briefly, RNeasy Mini kit was used for RNA isolation from γδ T cells or skin tissue according to manufacturer’s instructions. The extracted RNA was quantized by Nanodrop spectrophotometer (Invitrogen). RNA purity from 1.8 to 2.0 was considered as standard purity. RNA (28*S* and 18*S*) was also examined for quality, and RIN value no less than 7 was considered to be optimum by using Bioanzlyzer (Agilent Technologies, Santa Clara, CA). For reverse transcriptase PCR reaction, 50-ng total RNA was transcribed into cDNA using Reverse Transcription Kit (QIAGEN). RT-PCR was performed according to the manufacturer’s instructions and cDNA stored at −20°C till further use.

qPCR was then performed on RT-PCR detection system with SYBR Green Supermix (Bio-Rad). Primers were listed in **Supplementary Table S1**. Threshold cycle (27) values of target gene was normalized with housekeeping gene *Actb*.

### Adenoviral knockdown of *RORc*

Using RNAi Designer to construct an adenoviral RNAi vector (BLOCK-iT) (Thermo Fisher Scientific). This vector was transfected into 293A to produce adenovirus. The vehicle vector was used as a control. Dermal cell suspensions were obtained from leprosy lesions or healthy skin and activated with coated anti-CD3 and anti-CD28 for 24h. Adenoviral supernatants were harvested, filtered, and supplemented. Then infected γδ T cells were enriched through CD3^+^TCRδ^+^ sorting.

### Estimation of cytokines by ELISA

To detect the level of IL-17A, sandwich ELISA (Thermo Fisher Scientific) was used following the manufacturer’s instructions. In summary, 96-well plates (Thermo Fisher Scientific) were coated with 50 ml per well capture antibody and washed 3 times with washing buffer. The plates were then subsequently blocked with 1× assay diluent at room temperature for 1h. After blocking, the plates were washed 5 times with 1× washing buffer. A total of 50 ml per well of supernatant were added in triplicate and incubated for 2h at room temperature. After incubation, washing was done and 50 ml per well of detection antibody was added, and plates were further incubated at room temperature for 1h. A total of 50 ml per well of Avidin-HRP diluted in 1× assay diluent was added and incubated at room temperature for 30 min. Color development step was performed and the optical density was measured at 450 nm.

### Statistical analysis

The data were analyzed by Graph Prism 6.0 software (GraphPad, San Diego, CA), and the statistical significances were analyzed by unpaired Student’s *t* test, one-way analysis of variance (ANOVA), or two-way ANOVA. All quantitative data were shown as mean ± SEM. *P*-values less than 0.05 were considered significant.

## Data availability statement

The original contributions presented in the study are included in the article/**Supplementary Material**. Further inquiries can be directed to the corresponding author.

## Ethics statement

The studies involving human participants were reviewed and approved by Ethics Committee of Shanghai Skin Disease Hospital. The patients/participants provided their written informed consent to participate in this study.

## Author contributions

YL and DY designed the study; YL, CS, SM, and YM performed the experiments; YL, CS, SM, YM, XL, JZ, and DY analyzed the data; YL, CS, and DY wrote the manuscript. All authors contributed to the article and approved the submitted version.

## Funding

This study was supported by National Natural Science Foundation of China (No. 81872537).

## Conflict of interest

The authors declare that the research was conducted in the absence of any commercial or financial relationships that could be construed as a potential conflict of interest.

## Publisher’s note

All claims expressed in this article are solely those of the authors and do not necessarily represent those of their affiliated organizations, or those of the publisher, the editors and the reviewers. Any product that may be evaluated in this article, or claim that may be made by its manufacturer, is not guaranteed or endorsed by the publisher.
