# Supplementary material for: The protective role of tissue-resident interleukin 17A–producing gamma delta T cells in Mycobacterium leprae infection
Source: Front Immunol. 2022 Oct 26;13:961405. doi: 10.3389/fimmu.2022.961405 (PMC9644052; doi:10.3389/fimmu.2022.961405)
Supplement: Supplementary file 1 [file DataSheet_1.pdf]

Supplementary Fig.1

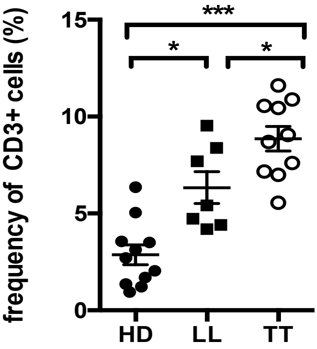

Supplementary Fig.2

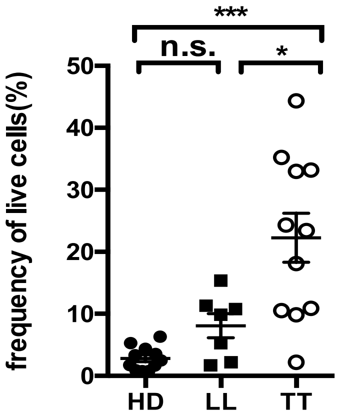

Supplementary Fig.3

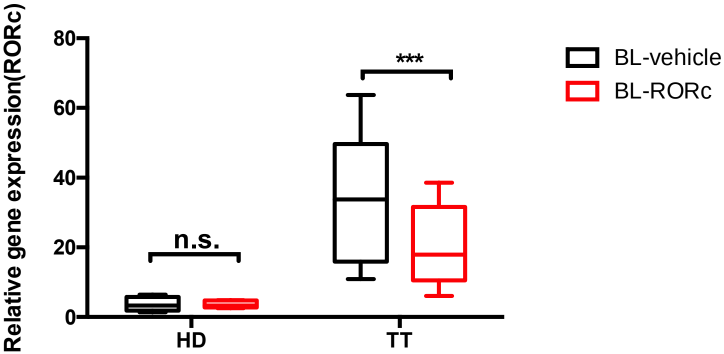

**Table S1. Primers for qPCR**

| Gene | Forward (5'-3') | Reverse(5'-3') |
|---|---|---|
| *Ngf* | GGCAGACCCGCAACATTACT | CACCACCGACCTCGAAGTC |
| *Ngfr* | CCTACGGCTACTACCAGGATG | CACACGGTGTTCTGCTTGT |
| *Il23r* | CAGGTCACTATTCAATGGGATGC | GCAGTTCTTAATTGCTGCTTGG |
| *Ccr1* | GACTATGACACGACCACAGAGT | CCAACCAGGCCAATGACAAATA |
| *Ccr2* | CCACATCTCGTTCTCGGTTTATC | CAGGGAGCACCGTAATCATAATC |
| *Ccr4* | CCCACGGATATAGCAGACACC | GTGCAAGGCTTGGGGATACT |
| *Ccr5* | TTCTGGGCTCCCTACAACATT | TTGGTCCAACCTGTTAGAGCTA |
| *Ccr6* | TTCAGCGATGTTTTCGACTCC | GCAATCGGTACAAATAGCCTGG |
| *Ccr7* | TGAGGTCACGGACGATTACAT | GTAGGCCCACGAAACAAATGAT |
| *Cxcr3* | CCACCTAGCTGTAGCAGACAC | AGGGCTCCTGCGTAGAAGTT |
| *Cxc4* | GGGCAATGGATTGGTCATCCT | TGCAGCCTGTACTTGTCCG |
| *Cxc5* | CACGTTGCACCTTCTCCCAA | GGAATCCCGCCACATGGTAG |
| *Il17a* | AGATTACTACAACCGATCCACCT | GGGGACAGAGTTCATGTGGTA |
| *Il22* | GCTTGACAAGTCCAACTTCCA | GCTCACTCATACTGACTCCGT |
| *Tnfa* | CCTCTCTCTAATCAGCCCTCTG | GAGGACCTGGGAGTAGATGAG |
| *Ifng* | TCGGTAACTGACTTGAATGTCCA | TCGCTTCCCTGTTTTAGCTGC |
| *Actin* | - CATGTACGTTGCTATCCAGGC | CTCCTTAATGTCACGCACGAT |
